# Identification of stable endogenous control genes for transcriptional profiling of photon, proton and carbon-ion irradiated cells

**DOI:** 10.1186/1748-717X-7-70

**Published:** 2012-05-17

**Authors:** Geeta D Sharungbam, Christian Schwager, Sara Chiblak, Stephan Brons, Lynn Hlatky, Thomas Haberer, Jürgen Debus, Amir Abdollahi

**Affiliations:** 1 Molecular RadioOncology [E210], National Center for Tumor Disease (NCT), German Cancer Research Center (DKFZ), Im Neuenheimer Feld 460, 69120, Heidelberg, Germany; 2 Heidelberg Ion Therapy Center (HIT), Heidelberg Institute of Radiation Oncology (HIRO), Department of Radiation Oncology, University of Heidelberg Medical School, Im Neuenheimer Feld 450, 69120, Heidelberg, Germany; 3 Center of Cancer Systems Biology, NASA Specialized Center of Research, St. Elizabeth’s Medical Center, Tufts University, 736 Cambridge Street [CBR 1], 02135, Boston, MA, USA

**Keywords:** Endogenous control genes, Internal control genes, qRT-PCR, Photon, Proton, Carbon-ion, Tumour cells, A431, A549 and BxPC3

## Abstract

**Background:**

Quantitative analysis of transcriptional regulation of genes is a prerequisite for a better understanding of the molecular mechanisms of action of different radiation qualities such as photon, proton or carbon ion irradiation. Microarrays and real-time quantitative RT-PCR (qRT-PCR) are considered the two cornerstones of gene expression analysis. In interpreting these results it is critical to normalize the expression levels of the target genes by that of appropriately selected endogenous control genes (ECGs) or housekeeping genes. We sought to systematically investigate common ECG candidates for their stability after different radiation modalities in different human cell lines by qRT-PCR. We aimed to identify the most robust set of ECGs or housekeeping genes for transcriptional analysis in irradiation studies.

**Methods:**

We tested the expression stability of 32 ECGs in three human cancer cell lines. The epidermoid carcinoma cells (A431), the non small cell lung carcinoma cells (A549) and the pancreatic adenocarincoma cells (BxPC3) were irradiated with photon, proton and carbon ions. Expression Heat maps, clustering and statistic algorithms were employed using SUMO software package. The expression stability was evaluated by computing: mean, standard deviation, ANOVA, coefficient of variation and the stability measure (*M*) given by the geNorm algorithm.

**Results:**

Expression analysis revealed significant cell type specific regulation of 18 out of 32 ECGs (*p* < 0.05). A549 and A431 cells shared a similar pattern of ECG expression as the function of different radiation qualities as compared to BxPC3. Of note, the ribosomal protein *18S*, one of the most frequently used ECG, was differentially regulated as the function of different radiation qualities (*p* ≤ 0.01). A comprehensive search for the most stable ECGs using the geNorm algorithm identified 3 ECGs for A431 and BxPC3 to be sufficient for normalization. In contrast, 6 ECGs were required to properly normalize expression data in the more variable A549 cells. Considering both variables tested, i.e. cell type and radiation qualities, 5 genes-- *RPLP0*, *UBC*, *PPIA*, *TBP* and *PSMC4--* were identified as the consensus set of stable ECGs.

**Conclusions:**

Caution is warranted when selecting the internal control gene for the qRT-PCR gene expression studies. Here, we provide a template of stable ECGs for investigation of radiation induced gene expression.

## Background

In addition to direct, e.g. DNA damaging effect, system level cellular responses to ionizing radiation are attributed to the initiation of intracellular signals and subsequent differential regulation of genes/pathways governing various cellular processes [1]. Therefore, detecting differential regulation of genes is critical for a better understanding of radiation-induced molecular effects. Transcriptional perturbation after cell exposure to different radiation qualities is investigated to unravel the systems biology of cellular response underlying, normal tissue toxicity, carcinogenesis, or anti-cancer effects of irradiation [1-3]. Therefore, these studies have ramification for a broad spectrum of basic and applied sciences ranging from effects of space radiation to carcinogenesis to cancer therapy.

In contrast to conventional photon irradiation the molecular effects of proton or heavier ions (e.g. carbon ions) are less explored yet. However, emerging data indicate molecular differences in transcriptional response of cells to particles as compared to photon irradiation [4,5].

One reliable and highly sensitive tool that allows rapid and accurate results in gene expression analysis is the qRT-PCR [6,7]. As in any gene expression analysis, selection of a valid normalization or endogenous control to correct for differences in RNA sampling is critical to avoid misinterpretation of results. Inter-sample variation due to sample collection, RNA preparation and quality, inherent sample differences, pipetting errors, different efficacies of the radiation qualities and reverse transcription efficiency are common sources of variability. The ideal endogenous control should have a constant expression level under different experimental conditions and be sufficiently abundant across different samples and cell lines. Although any gene that is stably expressed under a defined experimental condition can be used for normalization, the selection is most commonly made from the constitutively expressed ECGs.

However, the expression levels of the commonly used ECGs may not only vary in different cell lines but also under different experimental treatments or pathological states [8-25]. This necessitates the selection of ECGs which are appropriate for each experimental system. Although, there has been systematic selection of ECGs for various experimental systems, such selection has not been conducted so far for studying the effects of different radiation qualities.

Here, we investigate the expression stability of 32 commonly used ECGs in three human cancer cell lines irradiated with photon, proton and carbon ions. Differential regulation of ECGs was found as the function of both variables, radiation quality and cell type, respectively. Reliable internal control genes for individual cell lines were identified such as *PGK1, RPL37A* and *PSMC4* for A431; *RPLPO*, *UBC*, *GAPDH*, *MT-ATP6*, *CASC3* and *PES1* for A549*;* and *RPL37A*, *RPLPO* and *CASC3* for BxPC3. A systematic analysis further revealed 5 stable genes among the 32 candidate ECGs tested to normalize gene expression data generated in different cells and after various radiation qualities.

## Results

### Expression of the 32 ECGs

In this study, 32 ECGs (Additional file 1) were evaluated to identify the most suitable reference genes for gene expression profiling of irradiated cell lines. This collection of genes constitutes frequently used ECGs which were selected based on their relative high abundance and constitutive expression determined by literature search and/or whole genome microarray data. The three prototypic tumour cell lines-- A431, A549 and BxPC3 used in this study are among most commonly investigated model cell lines for each tumor entity. They were irradiated with photon, proton and carbon ions. After total-RNA isolation and quality control using lab-on-chip bioanalyzer, qRT-PCR was performed using Taqman primer and probes.

#### CT-values and ECG regulation

To get a better overview of the CT-values among all the cell lines, the CT-range along with the minimum and maximum CT-values were listed in Table 1. The variation of CT-values ranged from 8.97 in A431 to 28.58 in A549. The 8.97 CT-value corresponded to *18S* indicating its high abundance in the samples, whereas, the CT-value 28.58 corresponded to *GADD45A* indicating a moderate abundance. Moreover, *RPL37A, GAPDH* and *RPLPO* exhibited small CT-range indicating less variation in expression whereas the large CT-range of *ACTB, GADD45A, IPO8* showed large variations in their expression.

**Table 1:** Cycle threshold (CT) values and coefficient of variation (CV) of 32 endogenous control genes across the samples of the cell lines

**Gene Symbol**	**CT Range**	**CT Min.**	**CT Max.**	**Mean CT ± SEM**	**CV(%)**
*RPL37A*	1.13	17.95	19.08	18.44 ± 0.29	1.6
*HMBS*	1.39	23.96	25.35	24.61 ± 0.41	1.7
*CASC3*	1.22	22.79	24.01	23.45 ± 0.42	1.83
*RPLP0*	1.36	17.22	18.58	17.83 ± 0.34	1.91
*PSMC4*	1.56	21.04	22.6	21.90 ± 0.46	2.12
*ABL*	1.92	22.95	24.87	23.71 ± 0.51	2.15
*UBC*	1.62	19.22	20.85	19.81 ± 0.43	2.19
*GAPDH*	1.25	16.01	17.25	16.53 ± 0.37	2.27
*POLR2A*	2.03	23.5	25.53	24.19 ± 0.56	2.35
*MT-ATP6*	1.39	15.18	16.57	15.66 ± 0.38	2.46
*PES1*	1.97	21.04	23.02	22.12 ± 0.55	2.49
*TBP*	2.19	23.88	26.08	24.47 ± 0.64	2.62
*GUSB*	1.7	22.28	23.98	23.30 ± 0.63	2.71
*EIF2B1*	2.53	23.593	26.12	24.11 ± 0.70	2.91
*RPS17*	1.6	17.58	19.18	18.14 ± 0.53	2.92
*RPL30*	1.89	17.24	19.14	18.05 ± 0.53	2.96
*POP4*	1.17	22.73	23.91	23.64 ± 0.72	3.05
*PPIA*	2.15	17.43	19.58	18.08 ± 0.55	3.07
*PUM1*	2.88	22.15	25.03	23.10 ± 0.71	3.08
*HPRT1*	2.73	21.18	23.91	22.08 ± 0.68	3.1
*CDKN1B*	2.08	22.8	24.98	23.98 ± 0.81	3.41
*PGK1*	2.25	18.17	20.42	19.35 ± 0.69	3.61
*MRPL19*	3.09	22.73	25.82	24.01 ± 0.95	3.96
*CDKN1A*	2.6	21.3	23.99	22.98 ± 0.95	4.17
*ACTB*	5.65	16.1	21.75	17.80 ± 0.75	4.23
*ELF1*	3.59	21.81	25.39	22.76 ± 1.00	4.4
*IPO8*	4.26	23.68	27.93	24.80 ± 1.13	4.57
*GADD45A*	4.2	24.37	28.57	26.29 ± 1.37	4.97
*YWHAZ*	3.56	22.17	25.73	23.96 ± 1.26	5.27
*TFRC*	4.26	21.15	25.41	22.13 ± 1.18	5.37
*B2M*	3.76	17.99	21.75	19.33 ± 1.21	6.29
*18 S*	2.95	8.79	11.95	10.78 ± 1.02	9.5

The genes are sorted by the coefficient of variation increasing from top to bottom.

Four replicates were used for each cell line. Cell lines were irradiated with photon, proton and carbon ions. Non-irradiated samples served as control. Standard error of the mean (SEM).

**Figure 1:**
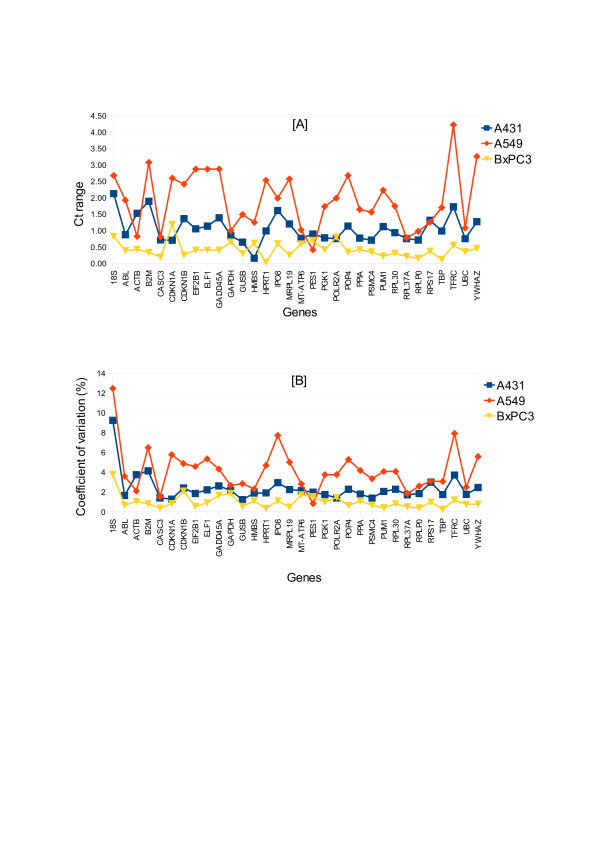
**Radiation induced variation in ECG expression. (A)** CT range and **(B)** coefficient of variation Figure shows the variations in the expression level of each ECG in A431, A549 and BxPC3. BxPC3 showed least variation as a function of different radiation qualities as compared to A431 and A549. A549 showed highest variations.

The CT-range and coefficient of variation for each cell line were listed in additional file 2. Within each individual cell line irradiated with photon, proton and carbon ion, all the ECGs in BxPC3 except for *CDKN1A, 18S, POLR2A, PES1* and *HMBS* with CT-range 1.18, 0.82, 0.80, 0.70 and 0.61, respectively, showed the smallest CT-range, while in A549 all the ECGs except *PES1*, *ACTB* and *RPS17* showed the largest CT-range (Figure 1A). In other words, as a function of different radiation qualities in BxPC3, the expression of the ECGs was considerably stable, while there was more variation in A549. Moreover, A431 and A549 shared similar expression pattern of the ECGs. The coefficient of variation plotted in Figure 1B also reflected the similar regulation of the 32 ECGs in the three cell lines.

#### ECG regulation using heat map

**Figure 2:**
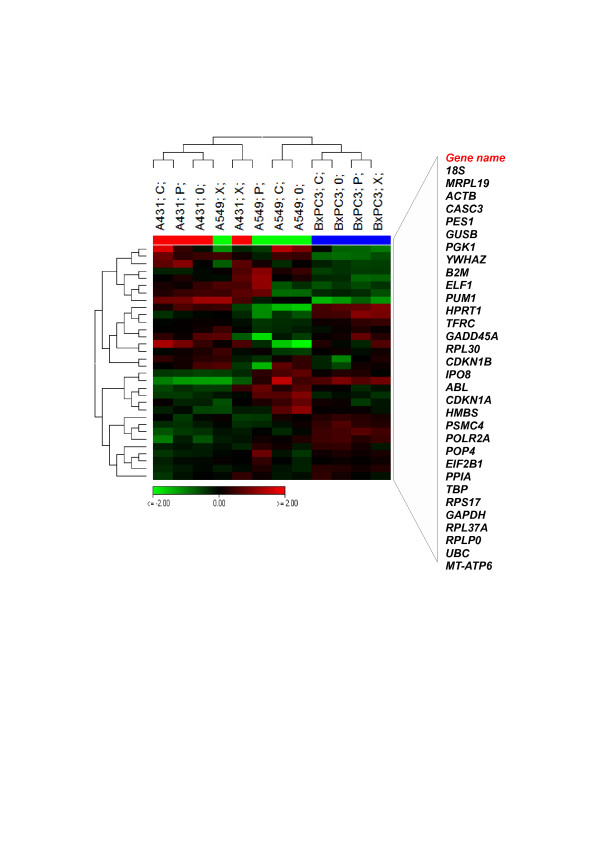
**Direct clustering of 32 ECGs expression.** This Heat map represents expression of all 32 ECGs across the three cell lines (A431, A549 and BxPC3) irradiated with photon, proton and carbon ion. Genes were hierarchically clustered by Pearson correlation coefficient using average linkage. Green denotes genes with relatively decreased expression while red denotes genes with relatively increased expression. Scale bar represent log_2_ expression level of ECGs. C = Carbon, P = Proton, X = Photon and 0 = Control. Expression profile of A431 and A549 are similar as compared to BxPC3 which showed unique expression profile with less variations among the samples.

The expression of the 32 candidate ECGs across the samples were also visualized in a Heat map (Figure 2). Direct clustering of the ECGs expression showed that the expression profile of A431 and A549 were more similar as compared to BxPC3. BxPC3 showed least variation whereas A549 showed maximum variation in expression among the samples as well as among the ECGs indicating a cell type specific expression of ECGs. As observed in Table 1, the Heat map also revealed low variation of *RPL37A*, *RPLPO* and *GAPDH* expression, while *ACTB*, *IPO8* and *GADD45A* depicted high variation in expression. The remaining ECGs were differentially regulated. For example, *B2M* and *ELF1* were up regulated in BxPC3 and A431 but down regulated in A549. On the other hand, *MRPL19* and *PES1* were down regulated in BxPC3 but up regulated in A431 and A549.

**Figure 3:**
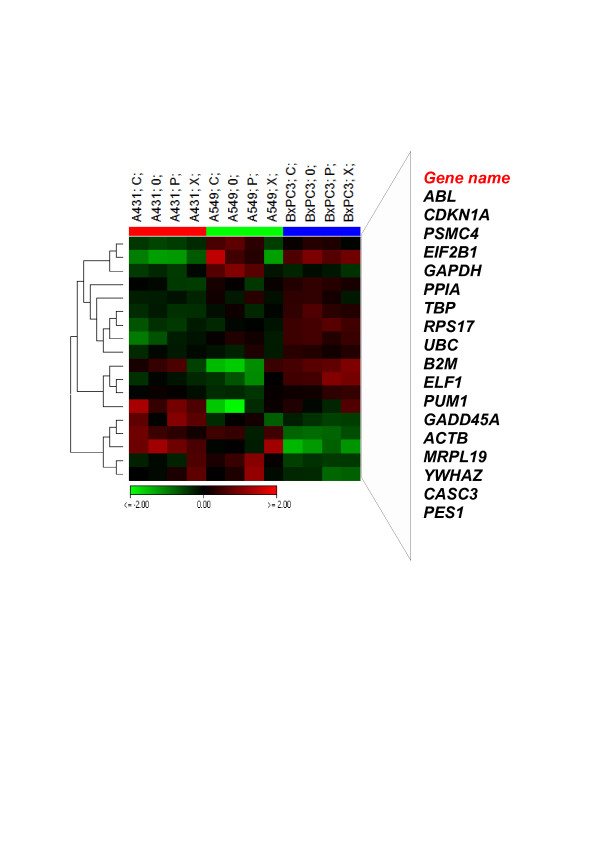
**Cell type specific regulation of ECGs.** This Heat map represents 18 ECGs which are significantly regulated according to ANOVA between the three cell lines, *p* < 0.05. Relative expression of each gene were normalized to the average intensity of the gene over entire samples (virtual pool). Green denotes genes with relatively decreased expression while red denotes genes with relatively increased expression. Genes are hierarchically clustered by Pearson correlation coefficient using average linkage. Scale bar represent log_2_ expression level of ECGs. C = Carbon, P = Proton, X = Photon and 0 = Control. Expression levels of the ECGs in BxPC3 is different as compared to A431 and A549. A549 showed maximum variation among the samples.

In Figure 3, we displayed a Heat map generated from a one way ANOVA analysis at *p < 0.05* between the three cell lines. It revealed 18 differentially regulated ECGs-- *ABL*, *CDKN1A*, *PSMC4*, *EIF2B1*, *GAPDH*, *PPIA*, *TBP*, *RPS17*, *UBC*, *B2M*, *ELF1*, *PUM*, *GADD45A*, *ACTB*, *MRPL19*, *YWHAZ*, *CASC3* and *PES1*. These could be cell type specific regulations as their radiation quality variation was minimal. Among them, the first 13 except for *PSMC4* were up regulated in BxPC3 and the remaining down regulated. In A431 and A549, 12 of them were down regulated and 6 were up regulated. This supports the finding that the expression levels of ECGs are different in BxPC3 as compared to A431 and A549. BxPC3 showed least variations and A549 showed maximum variations in gene expression as the function of radiation qualities. However, expression levels of ECGs were comparable in A431 and A549 (Figure 1A).

**Figure 4:**
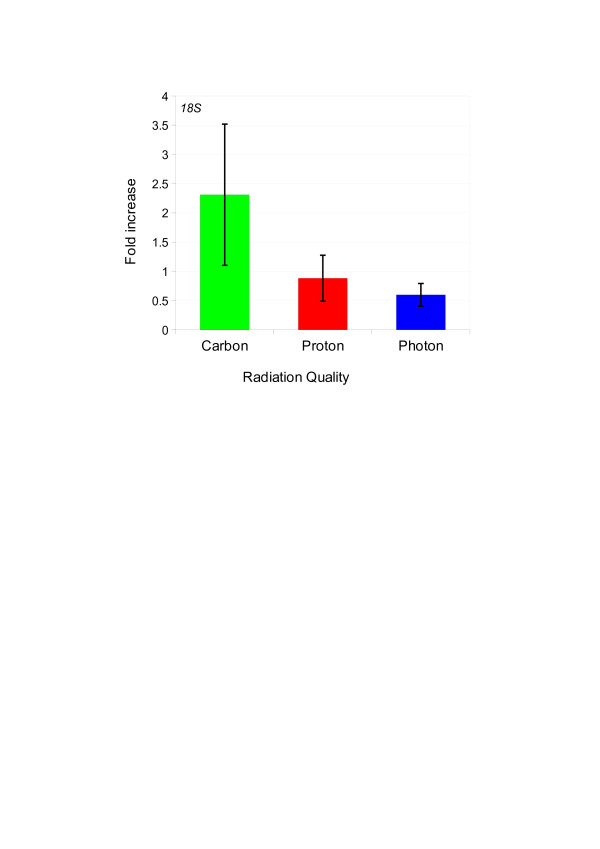
**Radiation induced differential regulation of ECG.** Figure displays gene expression levels of *18 S* in the three cell lines (A431, A549 and BxPC3) irradiated with photon, proton and carbon ion. Among the 32 ECGs, one gene; the ribosomal protein *18 S* was found to be differentially regulated as the function of different radiation qualities (*p* < 0.01 by ANOVA). Bars indicate mean expression ± standard deviation.

ANOVA analysis between the three radiation qualities at *p < 0.01* revealed one gene, the ribosomal protein *18S*, to be differentially regulated after different radiation qualities (Figure 4). This gene is one of the most commonly used internal control genes for normalisation of qRT-PCR based gene expression data. Therefore, caution needs to be practised in using this gene as an internal control gene, in particular when radiation effects are investigated.

The results in this section corroborated the findings of many other previous studies that the ECGs might be differentially regulated depending upon the experimental set-up and the cell type [8-25]. More importantly, this analysis seems to suggest that the ECGs are differentially regulated by the different cell types and radiation qualities. We attempted to confirm this observation employing a systematic analysis of the expression levels.

### Identification of appropriate ECGs

Gene expression levels obtained using PCR should be appropriately normalized by one or more carefully selected stable internal control genes. The geNorm algorithm developed by Vandesompele et al. [26] can determine the expression stability of control genes on the basis of non-normalized expression levels. This measure relies on the principle that the expression ratio of two internal control genes is constant in all samples regardless of the experimental condition or cell type. This algorithm computes a gene expression stability measure (*M*) for each gene based on the average pairwise expression ratio and then performs a stepwise exclusion of the least stable gene. Then the *M* values are computed again and stepwise exclusion performed until two genes are left. The genes with the lowest *M* values are considered to be the most stable across all the samples for each cell line.

#### Ranking of the 32 ECGs

The *M* values for all the 32 ECGs in A431, A549 and BxPC3 computed using the geNorm algorithm (integrated into SUMO software) were sorted and ranked in Table 2. This table revealed that the two most stable ECGs irrespective of the radiation qualities were: *PGK1-RPL37A* for A431, *RPLPO-UBC* for A549, and *RPL37A-RPLPO* for BxPC3.

**Table 2:** Control genes ranked in order of their expression stability*

**A431**	***M***	**A549**	***M***	**BxPC3**	***M***
*PGK1-RPL37A*	0.02	*RPLPO-UBC*	0.12	*RPL37A-RPLPO*	0.03
*PSMC4*	0.04	*GAPDH*	0.13	*CASC3*	0.03
*GUSB*	0.07	*MT-ATP6*	0.16	*RPL30*	0.08
*RPLPO*	0.08	*CASC3*	0.19	*UBC*	0.08
*UBC*	0.09	*PES1*	0.27	*RPS17*	0.08
*TBP*	0.12	*RPS17*	0.31	*EIF2B1*	0.10
*GAPDH*	0.17	*ACTB*	0.32	*ACTB*	0.12
*PPIA*	0.16	*TBP*	0.42	*POP4*	0.13
*ABL*	0.16	*PPIA*	0.39	*PSMC4*	0.13
*HPRT1*	0.17	*HMBS*	0.41	*PPIA*	0.13
*RPL30*	0.17	*PSMC4*	0.46	*ABL*	0.14
*PUM1*	0.19	*ABL*	0.51	*PGK1*	0.14
*ELF1*	0.20	*POLR2A*	0.54	*TBP*	0.14
*POLR2A*	0.22	*PUM1*	0.60	*GUSB*	0.15
*CDKN1A*	0.23	*RPL30*	0.64	*MRPL19*	0.17
*POP4*	0.28	*EIF2B1*	0.65	*CDKN1A*	0.17
*PES1*	0.32	*HPRT1*	0.66	*HPRT1*	0.18
*MT-ATP6*	0.33	*CDKN1B*	0.67	*PUM1*	0.20
*CASC3*	0.32	*MRPL19*	0.65	*HMBS*	0.21
*RPS17*	0.33	*RPL37A*	0.68	*MT-ATP6*	0.21
*HMBS*	0.34	*ELF1*	0.73	*B2M*	0.23
*EIF2B1*	0.37	*GUSB*	0.87	*GAPDH*	0.25
*YWHAZ*	0.39	*YWHAZ*	0.88	*PES1*	0.27
*CDKN1B*	0.40	*POP4*	0.92	*POLR2A*	0.27
*MRPL19*	0.43	*PGK1*	0.97	*YWHAZ*	0.32
*ACTB*	0.48	*GADD45A*	1.04	*ELF1*	0.33
*GADD45A*	0.51	*B2M*	1.01	*TFRC*	0.36
*IPO8*	0.52	*TFRC*	1.22	*IPO8*	0.37
*B2M*	0.52	*CDKN1A*	1.28	*18S*	0.41
*TFRC*	0.64	*18S*	1.27	*GADD45A*	0.45
*18S*	0.96	*IPO8*	1.34	*CDKN1B*	0.58

*M values increasing from top to bottom; the two most stable control genes in each cell type, for example *RPLPO* and *UBC* in A549, cannot be ranked in order because of the required use of gene ratios for gene-stability measurements.

**Figure 5:**
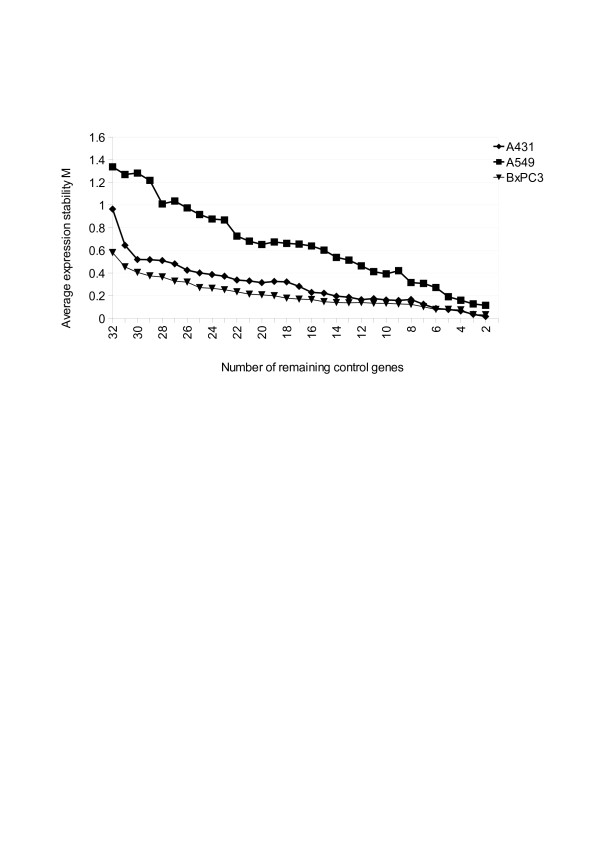
**Identification of the most stable ECGs in each cell line**. Most stable ECGs, i.e. not differentially regulated by different radiation qualities in each cell lines, are identified. Average expression stability *M* of all remaining control genes after stepwise exclusion of the least stable reference genes in three cell lines are shown. More stably expressed genes are positioned on the right side of the diagram, less stably expressed on the left side. ECGs are ranked in order of their expression stability and presented along x-axis. Stability values (*M*) determined by geNorm algorithm are presented along y-axis. Low stability value (*M*) reflects greater stability. For the gene names with their ranking refer Table 2.

The actual stepwise exclusion of the worst-scoring ECG was displayed in Figure 5. In this figure: (1) there was a very steep decrease in the average *M* value for A431 pointing at two unstably expressed ECGs, (2) the irregular decrease in the average *M* value for A549 showed that it has aberrantly expressed ECGs, (3) the regular decrease in the average *M* value for BxPC3 might mean that all the ECGs were stable.

#### Calculation of normalization factor

**Figure 6:**
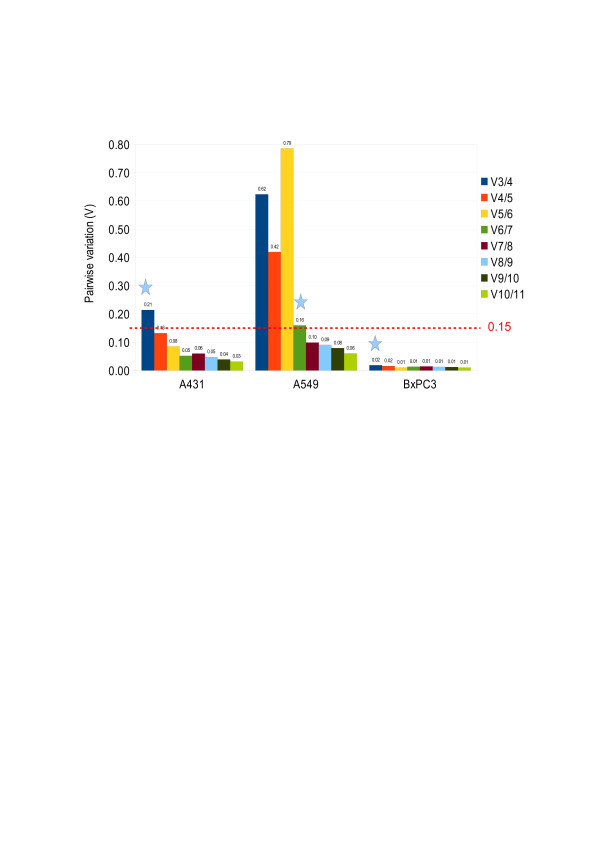
**Determination of the optimal number of control genes for normalization.** Pairwise variation (*V*_*n*(*n*+1)_) analysis between the normalization factors (*NF*_*n*_) and (*NF*_*n*+1_) to determine the number of control genes required for accurate normalization. Normalization factors were computed taking 3–11 most stable genes for all the three cell lines. Pairwise variation of 0.15 was taken as a cut off value [26]. For A431 and BxPC3 three ECGs were sufficient for normalization in contrast to six ECGs for A549.

For each cell line, the normalization factors (*NF*) were computed, first for the three most stable ECGs, by taking the geometric mean of their expression levels. This is followed by stepwise inclusion of the most stable remaining ECG. Then the pairwise variations *V*_*n*(*n*+1)_ were calculated for every series of *NF*_*n*_ and *NF*_*n*+1_, reflecting the effect of adding an (*n +* 1)th ECG (Figure 6) [26].

Figure 6 shows that the value of *V*_*3/*4_ was low for A431, implying that the first 3 ECGs (*PGK1*, *RPL37A*, *PSMC4*) were sufficient to be used for normalization. For A549, the low value of *V*_*6/7*_ indicated that the first 6 ECGs (*RPLPO*, *UBC*, *GAPDH*, *MT-ATP6*, *CASC3*, *PES1*) were sufficient for normalization. In BxPC3, the three most stable ECGs (*RPL37A*, *RPLPO*, *CASC3*) were sufficient for normalization purposes.

#### Validation of the gene-stability measure M

**Figure 7:**
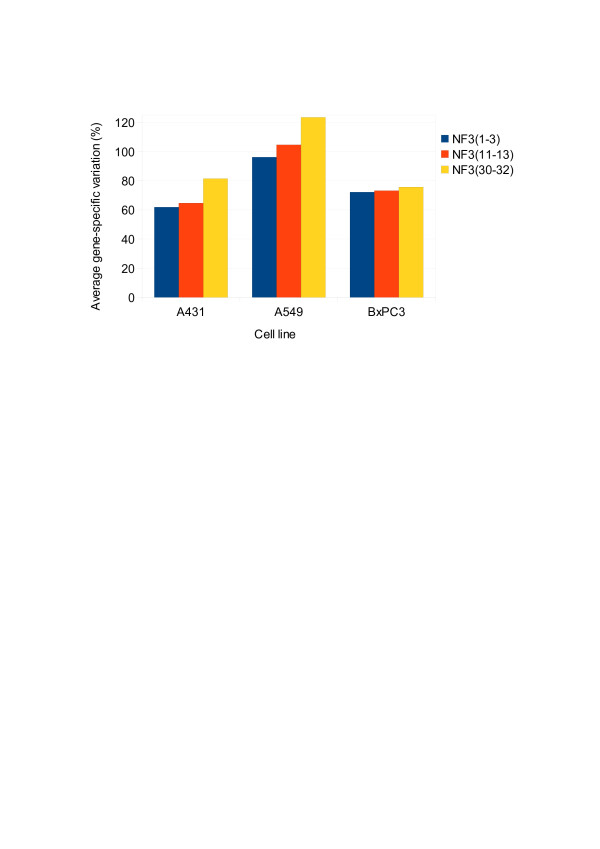
**Validation of the gene stability measure (*M*) and the geometric averaging of carefully selected control genes for normalization.** The average gene-specific variation (determined as coefficient of variation in percent) for the three control genes with the smallest variation within each cell line after normalization with three different factors calculated as the geometric mean of the three control genes with the lowest (*NF*_*3(1–3*)_), intermediate (*NF*_*3(11–13*)_) and highest (*NF*_*3(30–32*)_) gene-stability values (as determined by geNorm). The data confirmed the stability of the ECGs. The value under the star indicates the normalization factor for each cell line.

According to Vandesompele et al. [26], three different normalization factors were calculated based on the geometric mean of three genes with, respectively, the smallest *M* value (*NF*_*3(1–3*)_), the intermediate *M* value (*NF*_*3(11–13*)_) and the highest *M* value (*NF*_*3(30–32*)_) as determined by geNorm (Table 2). Further, we determined the average gene-specific variation of the three genes with the most stable expression (i.e., the smallest coefficient of variation) for each normalization factor within each cell line (Figure 7).It is conceivable that the gene-specific variation in all the cell lines were the least when the data are normalized to (*NF*_*3(1–3*)_). This validated that the gene-stability measure effectively identified the ECGs with the most stable expression.

#### Radiation-specific expression of the ECGs within the cell lines

**Figure 8:**
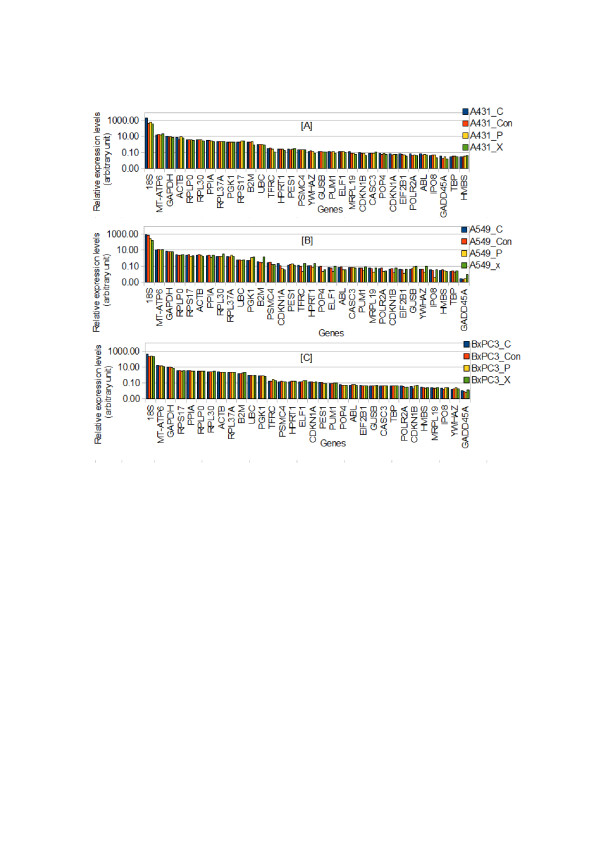
**Differential regulation of the ECGs after normalization with stable genes of each cell line**. **A)** A431 normalized by *PGK1*, *RPL37A*, and *PSMC4*; **(B)** A549 normalized by *RPLPO*, *UBC*, *GAPDH*, *MTATP6*, *CASC3* and *PES1*; **(C)** BxPC3 normalized by *RPL37A*, *RPLPO* and *CASC3*. As compared to the raw intensity data the differential regulation of the 32 ECGs are more pronounced after normalization with the identified stable genes for each cell line with correct normalization factor (*NF*_*n*_).

The gene stability measure *M* value was determined and validated, the candidate ECGs within each cell line were normalized by the appropriate stable ECGs found above and plotted in Figure 8. The variation in regulation of *18S* indicated that its expression depended upon the radiation quality (Figure 8A). This observation is in line with other above mentioned analysis performed (Figures 2 and 4). In addition, *GADD45A* was differentially regulated by radiotherapy in all three cell lines (Figure 8). Together, these data confirm differential regulation of candidate control genes as a function of different radiation qualities.

#### Identifying the consensus set of ECGs for comparative studies across all cell types

**Figure 9:**
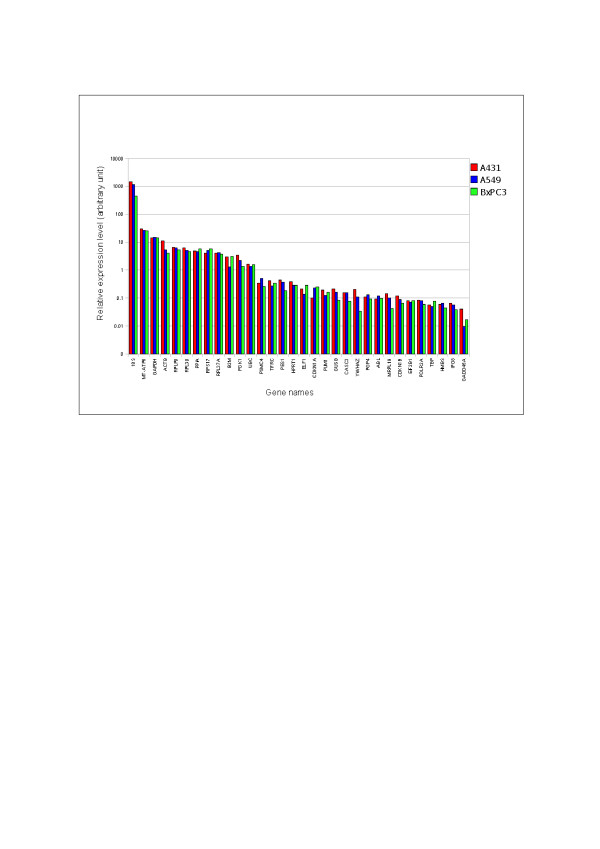
**Logarithmic histogram of the expression levels of 32 ECGs in all the cell lines.** The 32 ECGs were normalized to the geometric mean of five control genes (*RPLPO*, *UBC*, *TBP*, *PPIA* and *PSMC4*). In A431, a 422-fold expression difference is observed between the most stable gene (*PGK1*) and the least stable gene (*18 S*), while in A549 and BxPC3, a fold difference of 530 and 375 respectively were observed between them. In addition it shows the cell line specific differences in expression levels of particular genes (e.g. *YWHAZ*).

To compare the ECGs expression levels across A431, A549 and BxPC3 cells, first a consensus set of ECGs was identified for normalization of expression data using the algorithm suggested by Vandesompele et al. [26]. *RPLPOUBCPPIATBP* and *PSMC4* were selected by eliminating the ECGs with high *M* value to normalize and compare the cell type specific gene-expressions (Figure 9). Although the overall abundance of most ECGs among different cell lines was relatively similar, cell-line specific gene-expression were identified for some candidate ECGs such as, *18SB2MYWHAZPGK1CDKN1A* and *GADD45A*. In contrast, ECGs with a relatively constant expression included *GAPDHRPLPORPL30APPIAUBC* etc. In A431, a 422-fold expression difference was observed between the most stable gene (*PGK1*) and the least stable gene (*18S*) whereas a 530 and 375 fold difference in expression was found in A549 and BxPC3, respectively.

## Discussion

The emergence of a growing number of particle therapy facilities worldwide will stimulate comparative studies aiming to decipher the molecular mechanisms underlying differential biological effects of these novel radiation qualities. Comparative investigation of gene regulation on transcriptional level as the function of radiation treatment constitutes a cornerstone of these studies. Quantitative real time PCR (qRT-PCR) is considered the most sensitive method for detection of gene expression level. One limitation of this method is the need for proper endogenous control gene. To generate relative expression levels, the expression of the reference gene/s needs minimally alter among different types of cells or treatments. The goal of this study was to identify such ECGs.

We analysed the expression levels of 32 ECGs using the clustering, statistical methods such as ANOVA and the geNorm algorithm. Global analysis lead to the finding that gene expression profile in pancreatic cancer cells (BxPC3) is different as compared to the two other epithelial cancer cells tested i.e. epidermoid and lung carcinoma cells (A431 and A549). The ECGs in BxPC3 showed least variation in expression whereas A549 showed maximum variation in expression as the function of radiation qualities. Among the three cell lines, the ECGs were more stable in BxPC3. From the point of view of selecting appropriate ECGs this feature might be advantageous. On the other hand, it means that the ECGs in BxPC3 are less regulated by different radiation qualities.

ANOVA analysis of non normalized data revealed that 18 out of the 32 ECGs showed cell type specific differential regulation as shown by the differences in the expression profile between A431, A549 and BxPC3. In addition, significant radiation quality specific regulation was shown by one gene the ribosomal protein *18S*. Of note, this gene is one of the most commonly used internal control genes. Therefore, our data suggest validation of this gene prior to its use as internal control in radiation biology studies.

Although the clustering and ANOVA analysis of the raw data provided an overall overview and information about ECGs regulation, they do not allow the selection of appropriate stable ECGs for normalization of the qRT-PCR data. Next, the geNorm algorithm was employed to test for the stability of the 32 candidate ECGs as reference genes as well as selection of the optimal number of genes for normalization of gene expression.

Using the geNorm algorithm the two most stable ECGs for each cell line were determined- *PGK1-RPL37A* in A431, *RPLPO-UBC* in A549, and *RPL37A-RPLPO* in BxPC3. Further the optimal number of ECGs for the normalization of gene expression in each cell line was determined and validated: three ECGs each for A431 (*PGK1*, *RPL37A*, and *PSMC4)* and BxPC3 (*RPL37A*, *RPLPO* and *CASC3)* were recommended*.* In contrast, six ECGs for A549 data (*RPLPO*, *UBC*, *GAPDH*, *MTATP6*, *CASC3* and *PES1)* were required*.*

The normalized gene expression for each cell line in Figure 8 showed that *18S* and *B2M* are unstable genes under different radiation qualities. This is in contrast to earlier studies using *18S* and *B2M* as reference genes for proton therapy [4]. Besides, *PPIAACTB* and *UBC* for particle therapy using 0.5 Gy 4He ions in normal human lung fibroblasts [27] and *ACTB* for A549 [5] were reported as reference genes. However, Table 2 showed that these genes have intermediate stability within the cell line examined here.

Figure 8 demonstrates the regulation of particular genes treated with different radiation qualities. For instance, Figure 8B showed that in A549, *CDKN1A*-- a gene downstream of p53 pathway which is also implicated in regulation of cell growth and cell response to DNA damage-- is up regulated under all the radiation qualities, with maximum up regulation under photon. The gene *YWHAZ*-- involved in signal transduction by binding to phosphorylated serine residues on a variety of signaling molecule-- is up regulated in A431- photon while minimally regulated in A549-photon. *GADD45A*-- which binds to proliferating cell nuclear antigen, stimulates DNA excision repair in vitro and inhibits entry of cells into S phase-- was down regulated in A431 but up regulated in BxPC3. *18S*-- a component of the ribosome, the protein manufacturing machinery of all living cells-- is seen to be up regulated in carbon ion while its regulation varies for proton and photon in different cell lines.

In addition, five most stable ECGs (*RPLPOUBCTBPPPIA* and *PSMC4*) in three cell lines were selected as internal control genes for the normalisation of the gene expression independent of radiation qualities and cell type. This selection was based on the guideline of Vandesompele et al. [26].

The gene expression in each cell lines normalized by the selected five stable ECGs was shown in Figure 9. The expression of *ACTB* showed 2.8-fold difference between the highest and lowest expression levels, whereas *YWHAZ*, *18S*, *GADD45A* showed 5.8, 3.14 and 4-fold difference between the highest and lowest expression levels. The expression Heat map (Figure 3) also illustrate the regulation of *YWHAZ*, *GADD45A* and *18S*.

Selecting the ECGs for normalization across all the cell lines is a subtle issue. Although in [26], an algorithm to select the best ECGs within each specific cell line is presented, a clear method of selecting the best ECGs for all the cell lines is not given. More precisely, among the 5 selected ECGs (Table 3) -- *TBP, UBC, RPLPO, PPIA, PSMC4--* one ECG could be stable in one cell line, while it could be of intermediate stability in the other. However, our selection is supported by the fact that, in Table 2, all these genes are of intermediate stability in each of the cell lines.

**Table 3:** List of ECGs qualified as internal control genes across the cell and radiation qualities

**Symbol**	**Name**
*RPLPO*	Ribosomal protein, large, P0
*UBC*	Ubiquitin C
*PSMC4*	Protease 26S subunit, ATPase, 4
*PPIA*	Peptidylprolyl isomerase A
*TBP*	TATA box binding protein

## Conclusions

Careful selection and validation of ECGs prior to conducting radiation biology experiment is warranted. We report that different radiation qualities induced differential regulation of a number of ECGs among the candidate 32 “housekeeping genes”. Additional cell type specific gene expression was observed. Identification of the best internal control gene is a prerequisite for a successful quantitative measure of gene expression via RT-PCR. In this paper we provide a template for the identification of appropriate ECGs for the radiation induced gene expression studies. We identified reliable genes for individual expression profiling of the cell lines, the normalization of A431 may be done by *PGK1*, *RPL37A* and *PSMC4*; A549 by *RPLPO*, *UBC*, *GAPDH*, *MT-ATP6*, *CASC3* and *PES1;* and BxPC3 by *RPL37A*, *RPLPO* and *CASC3.* However, the 5 ECGs-- *TBP*, *UBC*, *RPLPO*, *PPIA*, *PSMC4*-- can be taken as the most suitable candidate reference genes for radiation response expression profiling in the tumor models studied. Moreover, this robust set of the most suitable candidate ECGs for radiation experiment may be applied and validated for the clinicopathological analysis of cancer specimens of epithelial tumors, non-small cell lung cancer and pancreatic adenocarcinoma.

## Methods

### Cell lines

The three different human tumour cell lines, i.e., the lung carcinoma cells (A549), the epidermoid carcinoma cells (A431) and pancreatic cancer cells (BxPC3) were used for the study. A549 and A431 cell lines were obtained from Deutsche Sammlung von Mikroorganismen und Zellkulturen GmbH (DSMZ) and BxPC3 from the American Type Culture Collection. The A549 and A431 cell lines were grown in 5 ml Dulbeccos Modified Eagle's Medium (DMEM) (Biochrom), BxPC3 was grown in 5 ml RPMI 1640 medium (GIBCO Invitrogen) supplemented with 10.0% FCS in T25 flasks (Becton Dickinson). Cells were cultured under standard conditions in a fully humidified incubator with 5.0% CO_2_ at 37.0°C.

### Irradiation

Cells were irradiated in T25 flasks with 2Gy of photon, 2Gy of proton and 1Gy of carbon ion. Photon was delivered by a linear accelerator at 6 Mev (Mevatron, Siemens, Erlangen, Germany). Particle irradiation with proton and carbon ion was done using a pencil beam in a spread out Bragg peak with 1.5 cm width equivalent to a depth of 14.0 cm in water, at the Heidelberg Ion Therapy Center (HIT) [28]. After irradiation, the cells were incubated for 12 h at 37.0°C. Control cells were treated identically but without irradiation. Cells were scrapped using the cell scraper after adding 300.0 μl TRIzol (Invitrogen) and collected in 1.5 ml Eppendorf tubes and subsequently stored at −20.0°C.

### RNA isolation and cDNA synthesis

RNA was isolated in phase lock tubes using TRIzol (Invitrogen) according to the manufacturer’s protocol. To avoid genomic DNA contamination RNA was treated with Dnase I (Ambion). Purified RNA was eluted in 20.0μL of nuclease-free water and stored at −20.0°C. RNA concentration and purity was assessed using a Nanodrop ND-1000 spectrophotometer (Peqlab). Integrity and concentration of RNA samples were determined by using RNA 6000 Nano Lab Chip kits and a 2100 Bioanalyzer (Agilent). RNA (2.0 μg) was subjected to reverse transcription reaction using the high-capacity cDNA reverse transcription kit (Applied Biosystems) according to the manufacturer’s protocol.

### Real-time PCR

To assess the expression of Human Endogenous Control gene set, real-time quantitative reverse transcription PCR (qRT-PCR) was performed on 32 candidate genes using TaqMan® chemistry (Applied Biosystems). Experiments were performed at least in triplicates for the three cell lines-- A431, A549 and BxPC3. Plates were run on a 7900HT Fast Real-Time PCR Systems (Applied Biosystems) using Fast 96-well blocks, Standard Fast PCR cycling protocol with 10.0μL reaction volumes. Cycling conditions used were-- 1 cycle initiation at 50.0°C for 2 min and 1 cycle at 95.0°C for 10 min, followed by amplification for 40 cycles at 95.0°C for 15 s and 60.0°C for 1 min. Amplification data were collected via Sequence Detection Systems 2.3 software (Applied Biosystems). The CT-values were computed with RQ Manager 2.xx (Applied Biosystems).

### Statistical analyses

Statistical analysis of data was performed using SUMO software package (http://www.oncoexpress.de/software/sumo). ANOVA was used to detect variation in the expression of the ECGs across the samples according to the radiation qualities and cell lines respectively. The average expression stability measure values (*M*) were computed using the geNorm algorithm suggested by Vandesompele et al. [26] (also incorporated in the SUMO program package).

## Competing interests

The authors declare that they have no competing interests.

## Authors contributions

GDS and AA designed the experiment, performed research, analysed data and wrote the manuscript. CS developed software and performed data analysis and statistics. SC, LH and JD analysed data and edited the manuscript. SB and TH performed the heavy ion irradiation planning and treatment. All authors read and approved the final manuscript.

## Supplementary Material

Additional file 1 List of 32 endogenous control genes used in the study. Click here for file

Additional file 2 **Cycle threshold range and coefficient of variation (CV) of 32 ECGs in each cell line.** The genes are sorted by the coefficient of variation increasing from top to bottom. Four replicates were used in all the three cell lines. Click here for file
