# Supplementary material for: Identification of stable endogenous control genes for transcriptional profiling of photon, proton and carbon-ion irradiated cells
Source: Radiat Oncol. 2012 May 17;7:70. doi: 10.1186/1748-717X-7-70 (PMC3422209; doi:10.1186/1748-717X-7-70)
Supplement: Additional file 1 — List of 32 endogenous control genes used in the study. [file 1748-717X-7-70-S1.pdf]

Additional file 1–Housekeeping genes.

Symbol	TaqMan® Assay ID	Name	Function
18S	Hs99999901_s1	18 S ribosomal	Ribosomal RNA
ABL1	Hs00245445_m1	Abelson murine leukaemia viral 1	Non-receptor tyrosine protein kinase
ACTB	Hs99999903_m1	Beta-actin	Cytoskeletal structure
B2M	Hs99999907_m1	Beta-2-microglobulin	Defense/immunity protein
CASC3	Hs00201226_m1	Cancer susceptibility candidate 3	Nonsense-mediated mRNA decay (NMD)
CDKN1A	Hs00355782_m1	Cyclin-dependent kinase inhibitor 1A	Regulation of cell growth and cell response to DNA damage
CDKN1B	Hs00153277_m1	Cyclin-dependent kinase inhibitor 1B	To stop or slow down the cell division cycle.
EIF2B1	Hs00426752_m1	Eukaryotic translation initiation factor 2B (Translation initiation factor eIF-2B subunit alpha)	Catalyzes the exchange of eukaryotic initiation factor 2-bound gdp for GTP.
ELF1	Hs00152844_m1	74-like factor 1 (ETS-related transcription factor Elf-1)	Protein, sequence-specific DNA binding, transcription activator activity and transcription factor activity.
GADD45A	Hs00169255_m1	Growth arrest and DNA-damage-inducible, alpha	Binds to proliferating cell nuclear antigen. Stimulates DNA excision repair in vitro and inhibits entry of cells into S phase.
GAPDH	Hs99999905_m1	Glyceraldehyde-3-phosphate dehydrogenase	Oxidoreductase, dehydrogenase
GUSB	Hs99999908_m1	Glucuronidase, beta	Galactosidase
HMBS	Hs00609297_m1	Hydroxymethyl-bilane synthase	Heme synthesis, porphyrin metabolism
HPRT1	Hs99999909_m1	Hypoxanthine guanine phosphoribosyl transferase 1	Glycosyltransferase
IPO8	Hs00183533_m1	Importin 8	Nuclear protein import
MRPL19	Hs00608519_m1	Mitochondrial ribosomal protein L19	Protein biosynthesis
MT-ATP6,LOC 100133315	Hs02596862_g1	Mitochondrially encoded ATP synthase 6	Produces ATP from ADP in the presence of a proton gradient across the membrane which is generated by electron transport complexes of the respiratory chain.

PES1	Hs00362795_g1	Pescadillo homolog 1, containing BRCT domain	Component of the PeBoW complex, which is required for maturation of 28S and 5.8S ribosomal RNAs and formation of the 60S ribosome
PGK1	Hs99999906_m1	Phospho-glycerate kinase-1	Catalyzes the reversible conversion of 1,3-diphosphoglycerate to 3-phosphoglycerate during glycolysis, generating one molecule of ATPX
POLR2A	Hs00172187_m1	DNA-directed RNA polymerase II subunit RPB1	DNA-dependent RNA polymerase catalyzes the transcription of DNA into RNA using the four ribonucleoside triphosphates as substrates.
POP4	Hs00198357_m1	Ribonuclease P protein subunit p29	Processing of precursor RNAs
PPIA	Hs99999904_m1	Peptidylprolyl isomerase A	Isomerase
PSMC4	Hs00197826_m1	Proteasome 26S subunit, ATPase, 4	Protease, hydrolase
PUM1	Hs00206469_m1	Pumilio, Drosophila, homolog of, 1	Protease, hydrolase
RPL30	Hs00265497_m1	60S ribosomal protein L30	Translation
RPL37A	Hs01102345_m1	Ribosomal protein L37a	Structural constituent of ribosome. Translation
RPLP0	Hs99999902_m1	Ribosomal protein, large, P0	Protein biosynthesis
RPS17	Hs00734303_g1	Ribosomal protein S17	Provides instructions for making a protein called ribosomal protein S17
TBP	Hs99999910_m1	TATA box binding protein	General RNA polymerase II transcription factor
TFRC	Hs99999911_m1	Transferrin receptor	Ion receptor
UBC	Hs00824723_m1	Ubiquitin C	Protein degradation
YWHAZ	Hs00237047_m1	Tyrosine 3-monooxygenase/ tryptophan 5-monooxygenase activation protein, zeta polypeptide	Signal transduction by binding to phosphorylated serine residues on a variety of signaling molecule
