# Supplementary material for: Identification of stable endogenous control genes for transcriptional profiling of photon, proton and carbon-ion irradiated cells
Source: Radiat Oncol. 2012 May 17;7:70. doi: 10.1186/1748-717X-7-70 (PMC3422209; doi:10.1186/1748-717X-7-70)
Supplement: Additional file 2 — Cycle threshold range and coefficient of variation (CV) of 32 ECGs in each cell line. The genes are sorted by the coefficient of variation increasing from top to bottom. Four replicates were used in all the three cell lines. [file 1748-717X-7-70-S2.pdf]

Additional file 2-Cycle threshold (CT) values range and coefficient of variation (CV) of 32 HKGs in each cell line

A431			A549			BxPC3		
**Genes**	**CT range**	**CV**	**Genes**	**CT range**	**CV**	**Genes**	**CT range**	**CV**
GUSB	0.64	1.23	*PES1*	0.41	0.83	*TBP*	0.12	0.25
CDKN1A	0.70	1.29	*CASC3*	0.81	1.52	*HPRT1*	0.03	0.35
PSMC4	0.71	1.39	*RPL37A*	0.77	1.84	*CASC3*	0.19	0.37
CASC3	0.71	1.40	*ACTB*	0.82	2.11	*RPLP0*	0.15	0.39
POLR2A	0.75	1.41	*HMBS*	1.25	2.32	*PUM1*	0.22	0.40
ABL	0.87	1.66	*UBC*	1.08	2.51	*MRPL19*	0.25	0.48
RPL37A	0.75	1.72	*RPLP0*	0.97	2.6	*RPL37A*	0.21	0.49
PGK1	0.78	1.74	*GAPDH*	1.00	2.67	*GUSB*	0.29	0.55
TBP	0.99	1.75	*MT-ATP6*	1.01	2.82	*EIF2B1*	0.40	0.55
UBC	0.75	1.76	*GUSB*	1.49	2.85	*POP4*	0.34	0.63
PPIA	0.77	1.79	*TBP*	1.70	3.07	*PSMC4*	0.35	0.66
RPLP0	0.71	1.84	*RPS17*	1.25	3.09	*ABL*	0.38	0.67
EIF2B1	1.05	1.86	*PSMC4*	1.56	3.34	*UBC*	0.35	0.75
HMBS	0.16	1.89	*ABL*	1.92	3.59	*YWHAZ*	0.46	0.78
HPRT1	0.99	1.91	*PGK1*	1.73	3.75	*B2M*	0.33	0.80
PES1	0.89	1.99	*POLR2A*	1.99	3.75	*RPL30*	0.31	0.81
PUM1	1.12	2.06	*RPL30*	1.74	4.08	*CDKN1A*	1.18	0.82
MT-ATP6	0.76	2.14	*PUM1*	2.23	4.08	*ELF1*	0.40	0.90
GAPDH	0.84	2.16	*PPIA*	1.64	4.18	*RPS17*	0.37	0.97
ELF1	1.13	2.22	*GADD45A*	2.87	4.32	*PGK1*	0.41	0.99
MRPL19	1.20	2.25	*EIF2B1*	2.87	4.58	*ACTB*	0.43	1.04
RPL30	0.93	2.28	*HPRT1*	2.53	4.70	*HMBS*	0.61	1.09
POP4	1.14	2.28	*CDKN1B*	2.41	4.87	*PPIA*	0.41	1.10
CDKN1B	1.36	2.4	*MRPL19*	2.57	5.02	*IPO8*	0.59	1.10
YWHAZ	1.27	2.45	*POP4*	2.68	5.29	*TFRC*	0.55	1.19
GADD45A	1.39	2.62	*ELF1*	2.87	5.35	*POLR2A*	0.80	1.46
IPO8	1.61	2.95	*YWHAZ*	3.25	5.58	*PES1*	0.70	1.53
RPS17	1.31	2.99	*CDKN1A*	2.59	5.77	*GADD45A*	0.40	1.67
TFRC	1.72	3.71	*B2M*	3.08	6.49	*MT-ATP6*	0.59	1.71
ACTB	1.52	3.75	*IPO8*	1.99	7.71	*GAPDH*	0.65	1.85
B2M	1.89	4.13	*TFRC*	4.22	7.91	*CDKN1B*	0.25	2.10
18S	2.12	9.24	*18S*	2.68	12.47	*18S*	0.82	3.79
